# The COVID-19 pandemic: local to global implications as perceived by urban ecologists

**DOI:** 10.1007/s42532-020-00067-y

**Published:** 2020-09-11

**Authors:** Ian Douglas, Mark Champion, Joy Clancy, David Haley, Marcelo Lopes de Souza, Kerry Morrison, Alan Scott, Richard Scott, Miriam Stark, Joanne Tippett, Piotr Tryjanowski, Tim Webb

**Affiliations:** 1 School of Environment, Education and Development, University of Manchester, Manchester, M13 9PL UK; 2 Lancashire Wildlife Trust, The Barn, Berkley Drive, Preston, PR5 6BY UK; 3 University of Twente, Enschede Area, The Netherlands; 4 Zhongyuan University of Technology, Zhengzhou, China; 5 Department of Geography, University of Rio de Janeiro, Rio de Janeiro, Brazil; 6 in-situ, Nelson, Lancashire, UK; 7 Complete Ecology Limited, 76 Tankerville Road, Streatham, London, SW16 5LP UK; 8 Director of the National Wildflower Centre, Eden Project, Bodelva, Cornwall PL24 2SG UK; 9 Department of Anthropology, University of Hawai’i at Mānoa, Saunders 346, 2424 Maile Way, Honolulu, HI 96822 USA; 10 School of Environment Education and Development, The University of Manchester, Manchester, M13 9PL UK; 11 Ketso Ltd, Stretford, UK; 12 Institute of Zoology, Poznań University of Life Sciences, Wojska Polskiego 71 C, 60-625 Poznań, Poland; 13 London National Park City, London, UK

**Keywords:** COVID-19, Urban ecology, Fieldwork, Funding, Environmental justice, Global south, Local knowledge

## Abstract

The global COVID-19 pandemic is affecting everyone, but in many different ways, stimulating contrasting reactions and responses: opportunities for some, difficulties for many. A simple survey of how individual workers in urban ecology have been coping with COVID-19 constraints found divergent responses to COVID-19 on people’s activities, both within countries and between continents. Many academics felt frustrated at being unable to do fieldwork, but several saw opportunities to change ways of working and review their engagement with the natural world. Some engaging with social groups found new ways of sharing ideas and developing aspirations without face-to-face contact. Practitioners creating and managing urban greenspaces had to devise ways to work and travel while maintaining social distancing. Many feared severe funding impacts from changed local government priorities. Around the world, the COVID-19 pandemic has amplified issues, such as environmental injustice, disaster preparation and food security, that have been endemic in most countries across the global south in modern times. However, developing and sustaining the strong community spirit shown in many places will speed economic recovery and make cities more resilient against future geophysical and people-made disasters. Significantly, top-down responses and one-size-fits-all solutions, however good the modelling on which they are based, are unlikely to succeed without the insights that local knowledge and community understanding can bring. We all will have to look at disaster preparation in a more comprehensive, caring and consistent way in future.

## Introduction

One of the key early responses to COVID-19 restrictions in towns and cities around the world was the increase in the use of parks and other greenspaces for exercise and recreation. Public awareness of the importance of urban greenspaces was heightened by people’s desire to escape into the open air. In Sweden, where soft measures centred around appeals to social distancing were implemented, rather than strict rules, people turned to urban nature (Samuelsson et al. 2020, p. 3). During the COVID-19 lockdown in Oslo, increases in urban greenspace use were greatest over trails within greener and more remote areas. Temporarily, green spaces probably acted as a substitute for prohibited indoor fitness and sport activities, and a refuge from stress during the COVID-19 lockdown (Venter et al. 2020, p. 8). In the UK at the start of the lockdown, people were advised that to relieve the stresses of isolation, they should maintain their relationship with nature, starting by spending more time parks, as long as they kept two metres apart (Mell, 2020). Many people involved in managing, restoring and safeguarding urban nature on the other hand were constrained in what they could do. The constraints included the need for social distancing; absence of colleagues who had to self-isolate; lack of volunteers who would normally have carried out maintenance and improvement tasks; diversion of local government staff to more pressing human health and safety tasks; and the inability to interact and confer with the people who would normally be visiting the parks, nature reserves and other urban greenspaces.

In this paper, we examine how individual workers in urban ecology have been coping with COVID-19 constraints and set people’s comments in the context of the global implications and likely consequences of the pandemic. In all considerations of COVID-19, the pandemic has to be seen as a global phenomenon in which critical situations can shift from region to region within a country, across a continent and around the world. The course of the pandemic will rely ultimately on global collaboration both to reduce the transmission between countries and to control infection rates within all countries rich or poor, peaceful or riven by conflict, political turmoil or other major disasters.

The heightened awareness of urban nature and its well-being benefits has been extolled by many recent books about how interaction with urban nature can improve human health. During the lockdown, book reviewers and journalists have written excitedly about the therapeutic values of gardening; the gains in mental health from interaction with plants; bird watching from windows (Muraweic and Tryjanowski 2020, p. 94); the tangible benefits of contact with nature; and the restorative theatre of nature (Clare 2020). This seeming re-assertion of long-established findings (e.g. Seymour 2003; Burls 2011; Matsuoka and Sullivan 2011; Tilt 2011) emphasises the wider notion that the pandemic has created both an opportunity and a need to address the environmental agenda and seriously tackle the climate crisis (Cavendish 2020, p. 12).

Enjoyment of nature in urban greenspaces is just one of the many ecosystem services provided by vegetation, wildlife, soils and water bodies in urban areas. Luederitz et al. (2015, p. 99) define urban ecosystem services as those services that are directly produced by ecological structures within urban or peri-urban areas. Such services include food production on urban farms, allotments or garden plots; but not food delivered from rural areas. Importantly, urban ecosystem services include all the climate modifying, pollution lowering, water flow regulation and temperature modifying characteristics of urban trees and other plants. Provision and maintenance of urban nature is essentially a continuous, multiscale socio-ecological practice: people engaging with nature to develop pleasing and valued greenspaces; and people engaging with other people to ensure that those greenspaces meet multiple needs and aesthetic desires (Xiang 2019, p. 12).

The United Nations Secretary General has suggested that we should use the recovery from the pandemic to build back better, not only socially and economically, but also by addressing urgent environmental and climate change concerns. This would include enhancing urban ecosystem services by increasing and maintaining urban greenspaces. The World Health Organisation (WHO) has emphasised that plans for post-COVID-19 recovery, and specifically plans to reduce the risk of future epidemics, should do more advanced preparation than just the early detection and control of disease outbreaks (WHO Europe 2020). Furthermore, such plans should lessen our impact on the environment to reduce the risk at its source including the illegal trade in wildlife (bushmeat).

### Use of urban greenspaces during the COVID-19 pandemic

Every country has imposed some form of COVID-19 restrictions, many of which led to reduced access to public spaces in urban areas. Throughout the UK, local governments had to inform citizens of changing park access regulations, but during fine weather, including a mini heat wave, parks were heavily used and social distancing became difficult. Opportunities were lost and gained. In London, the London National Park City programme had several projects, ready to launch, including one to protect woodland habitats on Streatham Common for future generations, that were delayed (Webb 2020). However, in both Edinburgh and Glasgow, Scotland, the city councils launched new online mapping tools so that people could share their suggestions for creating safer spaces in their city for walking, cycling and wheeling, as COVID-19 restrictions are eased (Greenspaces Scotland 2020).

In the USA, the City of Austin, Texas, made two responses to the pandemic: the establishment of a new “Conservation Corps” to create and care for trails, urban forests, native habitats and invasive species management and a “Healthy Streets” initiative for safe, socially distanced walking, bicycling, and other outdoor exercise on neighbourhood streets. Meanwhile, San Francisco, California, set up a “Find the Wild” project aiming to connect residents to the city’s parks, natural areas and urban wildlife.

Urban wildlife also has responded to reduced human presence in many parts of cities, with reports of sightings of pumas in downtown Santiago, Chile, of dolphins in untypically calm waters in the harbour of Trieste, Italy, and of jackals in broad daylight in urban parks in Tel Aviv, Israel. However, the pandemic has probably created new challenges for some species. Such urban-dwelling animals as rats, gulls or monkeys have become so dependent on food discarded or provided by humans that they struggled to find sufficient nutrition when people remained indoors. In countries where outdoor exercise was allowed during lockdowns, people going into urban greenspaces are likely to have disturbed resident wildlife (Rutz et al. 2020).

In the UK, the limitations imposed by COVID-19 lockdown have highlighted the need to be clever about improving access and creating pockets of green space to protect human well-being, and that of the wildlife returning to urban streets, parks and gardens (Collier 2020). Statements about the inequality of access to urban greenspace abound (Samuelsson et al. 2020; Plummer et al. 2020).

Such local actions are part of a global picture of increasing pressure on urban greenspaces, both those used for recreation and, particularly in most low-latitude cities, those used for urban food production. Individual experiences therefore have to be set in a global context, just as the COVID-19 pandemic has to be put in the global spatial and temporal context of vulnerability to disasters, conflicts, health emergencies, oppression, racism and victimisation.

To develop a deeper understanding of the complexity of the gains and losses of human interactions with and efforts to protect nature in the urban context during the pandemic, the UK Urban Ecology Forum (Box 1) asked the lead author to survey the views of both its members and of the authors of chapters in the forthcoming second edition of its Routledge Handbook of Urban Ecology (Douglas et al. 2020). Many of the Forum’s members are also contributors to the Handbook of Urban Ecology. The other contributors to the Handbook of Urban Ecology also come from a wide range of backgrounds and from countries in all continents. The editors had a deliberate policy to try to achieve a nationality and gender balance. Their experiences are governed by their particular expertise on aspects of the urban environment and by the cultures and practices of the places where they live and work. Several work outside their countries of residence and have been particularly constrained in what they can do by the lockdown regulations of more than one country. Here, we set out the global context and findings of this survey and provide examples of the individual responses that we received.

**Box 1:** The UK urban ecology forum

The UK Urban Ecology Forum is a network of people, including ecologists, artists, managers, environmental consultants, planners and researchers, involved with the environment and nature conservation in urban areas. It seeks to raise awareness; stimulate research; influence policy; improve the design and management of urban systems; and push urban nature conservation up the social and political agenda. It was established to provide advice to the nature conservation bodies of the four countries of the UK under the leadership of the late George Barker, formerly the Urban Nature advisor to English Nature (Natural England). It has produced influential guidance on urban greenspace policies and practices, particularly on standards for accessible natural greenspace in town and cities and on the need for standards that can help to achieve high quality adapted and attractive green spaces that people will want to use and will therefore help them get to know their neighbours and build stronger communities (see: https://urbanecologyforum.org.uk/)	

### Methods

The survey merely asked respondents to provide 100–150 words describing their current work, research or project and a further 100–150 words describing how their activity was changing, or was likely to change, as a result of the COVID-19 pandemic.^1^ Thirty-six UK Urban Ecology Forum members and 21 authors of Handbook chapters were contacted by e-mail. They were asked to complete a simple proforma and e-mail it back to the lead author. Thirty people (53%) responded. The chapter authors who replied came from ten different countries (excluding the UK) but with four from Europe, two from Asia, four from North America, and one each from Australia and Africa. Many of the respondents worked, or had, worked outside their own countries. Eighteen of the 30 respondents were academics, three were environmental artists, seven were practitioners on greenspace management or consulting, and two worked as urban nature advisers for the government nature conservation bodies of England or Wales.

Here, the general trends of these comments are reported under subheadings relating to the occupations of the respondents, for example: as academic researchers; practitioners working on nature reserves or publicly accessible greenspaces; consultants or landscape architects; or more general activists or campaigners. Parts of some responses are quoted verbatim, others are summarised. The individual remarks are complemented by comments from recent writings on the consequences of COVID-19.

A critical examination of the global context of these individual views then follows, with further reference to current writings on the impact of the pandemic. Differences of opinion and types of action being taken are used to emphasise and amplify the comments in a previous paper (Douglas 2020) arguing for greater attention to local and regional diversity and for tailoring responses to the pandemic to local situations using the knowledge and understanding of the people in local agencies, communities and public health systems.

## Researchers’ experiences during the pandemic

### Impacts of restrictions on academic fieldwork

Around the world, academic researchers have had their work cut short or interrupted by the lockdown restrictions imposed by the COVID-19 pandemic. Researchers whose activity involves fieldwork have been severely limited. Those working in foreign countries are particularly hard hit by quarantines and travel restrictions. Some have managed to obtain 1-year extensions from grant agencies, but important data may be lost from long-term ecological and environmental research programs. Even some locally based ongoing wildlife projects are being interrupted (Jacobus 2020). Some recognised longer-term opportunities. Chronicling sightings of unusual wildlife in quiet streets during lockdown, rigorously examining how animals’ behaviours change as people leave city centres, and then investigating what happens once humans begin re-populating cities would not only help us answer fundamental questions in animal behaviour and urban ecology, but also help us rethink how wildlife can live within our cities (Diamant et al. 2020).

For those engaging with communities, life became more difficult. Foreigners might be seen as carriers of the disease. One researcher reported that, during COVID-19, she had to fundamentally change her research methods to ensure physical distancing for the safety of research participants. She adapted her methods to replace face-to-face interviews and became more sensitive to the emotional language of the people she was studying (Lin 2020). If there is any good thing to emerge from this pandemic, it could be our ability to rethink risks, security, uncertainty and improvising communications and deeper thoughts about ethics (Lin 2020). These are vital skills for researchers as we will inevitably continue to live with viruses.

### Views on opportunities lost and new ways of operating expressed in the UK Urban Ecology Forum survey

Academic researchers responding to the UK Urban Forum frequently stated that their fieldwork had ceased entirely during the lockdown as universities had restricted research and travel; cessation of construction work had prevented data being collected from new field experiments; social distancing requirements had prevented face-to-face meetings; and many public sector collaborators had been diverted to more pressing tasks. The sections in quotation marks below are verbatim extracts from the replies to the survey.

One of those unable to travel to her work in SE Asia, anthropologist Miriam Stark, commented that her “Archaeological fieldwork was cancelled, which gives us time to explore and write about resilience strategies in historical responses to climate change in the region. New opportunities that we hope will open up include collaborations with conservation and climate scientists, both to deepen our understanding of long-term climate records for the Lower Mekong, to build a richer database of the region’s wetland fauna and how it changed through time”.

Others found new opportunities to ascertain how people sought new ways to enhance their social and psychological resources through contact with nature. They asked questions about changes in the use of urban greenspaces due to social distancing and COVID-19 restrictions. A landscape architect saw the pandemic opening new opportunities for the landscape profession to care about resource optimization as a core strategy in the design and management of urban greenspaces. Others reminded us that although the pandemic had led to a reduction in some urban environmental hazards, we must also think about climate-related events like heat waves which might increase risks for people living in highly built-up areas, with no private garden space and few opportunities to access good quality greenspaces.

Ornithologist Piotr Tryjanowski commented “I see a lot of potential opportunities. Lockdown is affecting people, but how is it affecting birds? There are possible different scenarios: one is back to wild, the other lack of habituation, because fewer people are around”. Where good comparative data are available for previous years, valid comparisons can be made (Morelli et al. 2018, p. 91).

Participatory planning researcher, Joanne Tippett, found ways of continuing her work with Ketso (a “workshop in a bag” that promotes productive collaboration in group meetings, training and for community engagement*)* by conducting remote workshops: “The pandemic has shown that rapid change is possible, and opened a space of opportunity to reimagine our urban spaces and relationship with the natural world. The need for meaningful engagement has never been more important. We will need to harness the ingenuity of communities and those working across all sectors to realise the potential to “build back better”. We need to make sure that everyone is heard in this dialogue, to have a better change of building an equitable and fair future. We have been trialling new approaches to engagement, to maximise the value of the hands-on, shared visual language of Ketso in remote workshops. This has required a shift in thinking in the Ketso team, as we have spent decades developing tools for people to build their ideas together in the same physical space. We are looking forward to rolling out this new approach to help deepen and widen the dialogue about the future”.

Undoubtedly, the great attention to the continuing and emerging COVID-19 situation is permeating academic enquiry (Hulme et al., 2020; Malanson 2020; Santiago-Alarcon and MacGregor-Fors 2020) and provoking many thoughts about how green recovery programs might support urban greening, sustainability and the mitigation of global heating. Social scientists working in other countries can use this very difficult time to pilot new research methodologies that could ultimately increase parity with their host-country partners because only the host-country partners can now do fieldwork, and working towards greater parity with host-country partners promotes a kind of global research resilience. However, the success and survival of the greenspaces we already have will depend on those directly for creating and managing them.

## Views expressed on the effects of the pandemic by those responsible for nature reserves and protecting urban nature

### Engaging with local communities

Practitioners involved in the survey ranged from those working in local greenspaces to others developing national strategies for urban nature and environmental artists. Survey responses included two people directly managing urban nature reserves and greenspaces in Greater Manchester, UK. Mark Champion, Wigan Projects Manager for the Lancashire Wildlife Trust, had responsibility for some 1500 ha of “nature reserve” and engagement with local communities. He wrote that under COVID-19 restrictions “Community work has been severely reduced and there is little formal consultation and capacity development. Arts projects have been curtailed. Citizen science recording of wildlife has been suspended, as have our training courses for volunteers. However, conservation work is continuing with several projects involving capital investment being completed. Surveys led by staff members have had to be modified to meet UK government COVID-19 safety criteria”. Later, in July, he commented: “As the lockdown was released in the UK there seemed to be a release of pent-up energy or cabin fever that, coupled with exceptionally good weather, led to a temporary but large rise in anti-social behaviour, with illegal raves and littering. Among the most obvious was a site at Wigan from which we removed nearly 5 tonnes of rubbish after a one-night illegal party. Fly-tipping, the illegal dumping of waste material, rubbish and rubble on roadsides, farm entrances, and open land in peri-urban areas, was a problem too. This was reported widely from a range of locations, many of which were not as urban as Wigan. The RSPB (Royal Society for the Protection of Birds) reserve at Haweswater in Cumbria, which is as rural at it is possible to get in England, was quite badly hit”.

Richard Salisbury, neighbourhood engagement officer for Manchester City Council at the Chorlton Water Park in the Mersey Valley south of the city, noted the impact of the halting of practical volunteering: “This is having an impact on conservation and some areas are becoming undermanaged. The adverse impacts if this continues would involve scrub taking over sensitive areas of reed beds and grasslands. Also, invasive species, such as Himalayan balsam, which we usually remove, could get a foothold in new areas”. All activities in parks and community meetings were cancelled, but it was hoped that eventually video meeting and social-distanced park activities could be organised.

People working as ecological and landscape consultants faced problems of not being able travel, also noting that while online meetings have expanded rapidly in Europe and North America, many overseas cultures are still deeply rooted in face-to-face meetings. Many clients had closed down the majority of their construction sites and progress on planning and design was slowed because local authority and regulator response times increased during the lockdown. Alan Scott, who runs his own company “Complete Ecology Ltd.” had all his work stopped by the lockdown and had to furlough all his staff. He thought that “The challenge will be to work and travel whilst maintaining social distancing. Suitable risk assessment and safety precautions will hopefully overcome this. The longer term however is more of a problem as many of our clients will be short of funds and may see other issues as a higher priority”.

Three environmental artists had differing responses. In Japan, Patrick Lydon had ecological art exhibitions in Kyoto cancelled just 2 days before they were due open. However, within a few days, Lydon’s team had built an online platform and had created an online audience, thanks to The Nature of Cities’ new virtual galleries at the Urban Ecological Arts Forum (https://www.thenatureofcities.com/friec/japan-queens-2020/). The 700 people who attended the virtual opening would not have been able to fit physically into the gallery in Kyoto.

David Haley was already working on critical recovery from disasters in Cumbria, UK, seeking to enable people to learn for themselves how to change from being victims into survivors and from survivors to proactive, self-determined, participants in ecologically resilient societies. He saw disasters, such as the COVID-19 pandemic, as bringing opportunities as well as danger: a paradox which might enable communities to generate creative cultures of resilience that would help in addressing major issues such as the climate emergency and extinction crisis.

Kerry Morrison had been working on a project to raise public awareness of the Pendle Hill Peat Restoration program in Northern England. With a local cook, she developed a vegetarian “Peat Pie” using brown lentil dhal to represent peat, with the aim of selling it to local eateries. A launch was planned for mid-June 2020, coupled with a mass peat seeding community planting event as a prelude to the launch. The project was championing the mantra: act local, think global. Kerry and her colleagues are hoping to launch the Peat Pie in the spring of 2021 and to reach out other communities for more recipes that address the climate emergency and celebrate diversity.

### Wider implications for those engaged in actively promoting, creating and restoring urban nature

**Fig. 1:**
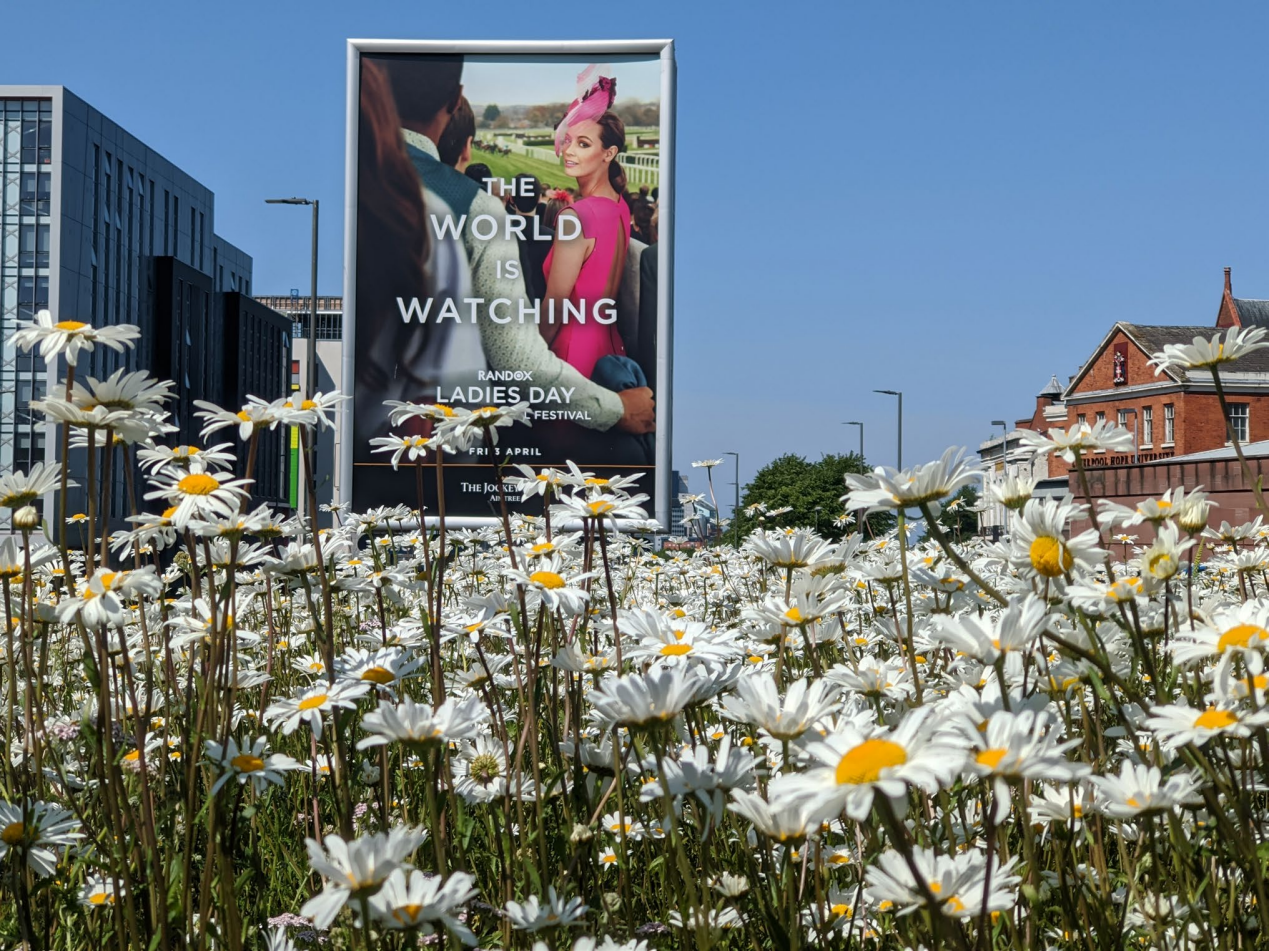
Nature on the doorstep: wildflowers in Liverpool, UK (Photo: Richard Scott)

Right now, during the pandemic, Richard Scott, Director of the UK National Wildflower Centre within the Eden Project, argues is the time to press forward with practical solutions that show love and respect for nature in building sustainable futures by working to meet social and cultural needs through safeguarding and enhancing nature on our doorsteps (Fig. 1). He wrote: “Right now it is important to demonstrate the carbon capture ability of all habitats, it is not just planting trees, it must be mosaics of habitats, which respect soil, both as a resource for food production, but also recycling unproductive urban substrates, for biodiversity and conservation. … We must be bold and imaginative and use inclusive language in a world desperate for positive futures now”.

Tim Webb, a Trustee of London National Park City, a roots-up organisation with an ambition to make London, and other cites, greener, healthier and wilder, had begun to set up a park ranger programme and had switch to virtual meetings and reorganise planned events. During the lockdown “In mid May 2020, our Rangers were planning a series of interventions, using digital technology, word of mouth, music and art to engage Londoners in new ways with life in a National Park City. We are rewilding Londoners, re-wiring brains to perceive our built environment as an enhanced natural landscape of mosaic habitats, all interlinked and waiting to be explored on foot, by bicycle and, at some point in the future, by public transport!”. Tim further argues that “The explosion of mutual aid groups witnessed during the COVID-19 outbreak is evidence of strong community spirit. Encouraging that spirit will speed out economic recovery and make cities more resilient against biological, ecological, meteorological, economic civil or political strife”.

### Critical aspects of the impact of COVID-19 on deprived people in all societies: energy and food security

Several Urban Ecology Handbook authors were concerned with the impacts of COVID-19 on human well-being, energy and food scarcity and wider environmental justice. People foresaw the looming potential food security disaster in Africa and the consequences of both local and global disparities, not only in income but also in access to assistance. Respondents asked whether the COVID-19 pandemic had heightened the need for more urban and peri-urban food growing, both by individual households and as commercial food production, in order to assure sustainable food supplies, both in emergencies and over the long term.

At the global scale, the World Bank saw the primary risks to food security during the COVID-19 crisis being within countries through disruptions in domestic food supply chains; problems in food production, harvesting and processing; and loss of incomes and remittances. The United Nations World Food Programme warned that crises in household finances could leave 265 million people facing acute food insecurity by the end of 2020, compared to the 135 million people at risk before the crisis (World Bank 2020).

#### Energy poverty and food security in Europe

Food security frequently affects people who live in energy poverty. Energy poverty arises when people have insufficient access to, or ability to buy, the fuel or electricity they require to meet their basic needs for cooking, heating or cooling, or other aspects of domestic life (https://ec.europa.eu/energy/eu-buildings-factsheets-topics-tree/energy-poverty_en?redir=1). Gender and energy specialist Joy Clancy has worked intensively on what can be learnt from the Global south concerning gender and energy poverty. This experience led her to write: “COVID-19 has an influence on demographics and on income, both of which are factors in energy poverty. Women in the retirement age group in Europe, due to a gender earnings gap at an earlier stage in the life cycle, are more like to live in income poverty than men. This situation is exacerbated by women’s longer life expectancy than men. Male mortality as a consequence of viral infection seems to be significantly higher than for women. Age also is a factor. The increased viral mortality of older men leaves a larger group of women without sufficient income to pay energy utility bills. In terms of those in employment, women dominate the service and retail industries in which job losses have been high. We need to know how these effects are influencing energy poverty and whether the measures taken to address the effects of the virus also prevent people experiencing (enhanced) energy poverty”.

In Europe, as elsewhere, high rates of energy poverty correlate with higher-at-risk-poverty rates, food poverty and sometimes ill-health (Omic 2019, p. 1). During COVID-19, the growing European severity of poverty has involved both energy and food insecurity. In the second quarter of 2020, multiple issues came together to cause tens of thousands of tonnes of produce to rot in fields, with farmers’ incomes falling while hunger among jobless workers soared (Fleetwood 2020). The continent’s heavy reliance on complex supply chains, imported food and just-in-time delivery all contributed to problems during COVID-19.

By June 2020, more than 100,000 people in the UK, who cared, without pay, for older, disabled or seriously ill relatives, had been forced to use food banks during the COVID-19 pandemic. As elsewhere, rising unemployment is beginning to add to the difficulties faced by deprived households. Such situations have shown how fragile the UK’s emergency food aid system is (Power et al., 2020, p. 3). Before the crisis, food banks (and other community food providers) received food in three ways: redistributed surplus food from supermarkets or charities; food donated by individuals; and food purchased in bulk from local supermarkets and shops. These sources of supply have been disrupted during the social and economic crisis surrounding COVID-19. Individual food donations fell as households prioritised their own food supply, while many food banks could not obtain the non-perishable items required for standard food parcels, due to the rush by families to stockpile things like toilet rolls and tissues at the start of the crisis in March 2020. Supermarkets faced temporary shortages and resorted to rationing such items.

#### COVID-19 and food security in the global south

The COVID-19 pandemic has amplified food security issues that have been endemic in the vast majority of the countries of the South in modern times (Zurayk 2020, p. 18). The modern globalised food system works in ways that control what, when, and how people eat by dominating producers, suppliers and retailers. The net effect is to exacerbate obesity and undernutrition and non-communicable diseases related to poor diet or inadequate food.

In Addis Ababa, Ethiopia, where the food situation deteriorated from April to June 2020, a telephone survey revealed that COVID-19 pandemic had adversely affected the majority of households in the city (Abate et al. 2020, p. 6). Over 66% of respondents indicated that their incomes were lower than expected in May 2020 and 45% reported that they had become extremely stressed about the ongoing situation. Less wealthy households were disproportionally affected.

In such deteriorating situations, critical aspects of the food system may cease to function effectively. One of these critical aspects is that at least half of Africa’s households rely on informal food vendors (Wegerif 2020, p. 798). In South Africa, itinerant and informal traders were forced to stop all activities during the lockdown. Families had to travel by public transport to get to the supermarkets that remained open. This increased the risk of spread of the virus, as supermarkets became one of the main sites of COVID-19 transmission in South Africa. Despite some traders obtaining permits to start selling again, the food system had been disrupted. So important are these small-scale, owner-operated, enterprises for the local food system that they should be supported to reach their full potential in normal times and be able to operate effectively during future emergencies. Such support will have to avoid disrupting their modes of operation and their economic and social benefits (Wegerif, 2020, p. 800).

Urban ecology has an important role to play in alleviating the urban food security crisis. Although world cereal production increased by 2.3% from 2018 to 2019, the potential doubling of the number of people at risk of food insecurity by the end of 2020 poses a global challenge. Urban agriculture may help to meet this challenge (Lal 2020). Urban agriculture comprises all forms of agricultural production (food and non-food) occurring within or around cities. It contributes to many ecosystem services, such as improving human health, and food access for local communities. It can provide income and jobs, enhance aesthetic value and beauty and raise community resilience. Urban agriculture can be a form of urban greenspace, whether in gardens, dedicated allotments or on temporarily unused land, with CO_2_ uptake by growing plants helping to reduce greenhouse gas emissions. Great expansion of urban food production is possible. For example, in the city of Sheffield, UK, there is more than enough land to meet the fruit and vegetable requirements of its population (Edmondson et al. 2020). During COVID-19, informal evidence suggests that people fortunate enough to have their own suburban gardens were turning increasingly to vegetable growing. However, at the global scale, provision of land or other opportunities, such as on rooftops, for food growing in expanding cities should become standard practice.

## Dealing with aggravated environmental and social injustice

Inequalities of both vulnerability to infection and of access to care during the COVID-19 pandemic are linked to wealth and race (Dorn et al. 2020; Higgins-Desbiolles 2020; Teixeira da Silva 2020). In both the USA and the UK, early reports indicated that COVID-19 rates of infection were higher among particular ethnic minorities (Khunti et al. 2020; Laurencin and McClinton 2020). In the UK Urban Ecology Forum survey, marked differences in the amounts of accessible greenspace in urban areas between high- and low-income areas were frequently noted. Marcelo Lopez de Souza, from Rio de Janeiro, Brazil, commented that during COVID-19 “The interface between environmental injustice and public health is becoming even more evident”. He has previously written about the “bourgeois environmentalism” that sees informal settlements as harmful to the environment and therefore should candidates for demolition. However, people in such settlements should have as much right as all other urban residents to accessible greenspace (Lopes de Souza 2016).

Above the recognisable injustice in terms of local urban ecosystem access and use, there is the injustice in the damage to both urban and rural ecosystems caused by conflicts and wars. The greatest injustice is probably the way the innocent victims of long-lasting conflicts are being affected by the COVID-19 pandemic. For example, cases of COVID-19 arose in early July 2020 among medical staff at Bab al-Hawa Hospital in Idlib, Syria. Here, only 600 physicians are left to care for over 4 million people (equivalent to about 1.4 medical doctors per 10,000 people). In addition, there are just 0.625 hospital beds per 1000 people, 5.7 intensive care unit beds per 100,000 people and only 47 functioning adult ventilators for the whole region (Ekzayez et al. 2020). This unjust deprivation of adequate health care is likely to hamper the effective control of COVID-19 in the long term.

In conditions possibly even more severe than those in Idlib, before the pandemic Yemen was experiencing the world’s worst humanitarian crisis with 5 years of war leaving over 24 million people needing life-saving support. Since March 2020, low testing rates for COVID-19 in Yemen have made it extremely difficult to know exactly how many cases there are, let alone track, trace and isolate people with infection for effective control (Ivers and Walton 2020). One estimate suggests that up to 11 million people could be affected with some 72,000 deaths (Looi 2020). Nearly 100 medical staff in Yemen had died of COVID-19 by 25 July 2020 (Associated Press 2020). Such pressures add to the problems caused by floods, desert locusts, economic turmoil and fighting that have already aggravated the food security situation in Yemen.

The COVID-19 pandemic requires a global response for vulnerable populations in countries like Syria and the Yemen. In such situations, good testing and subsequent isolation procedures, keeping families together if necessary, must be fully operational (Nott 2020). Because COVID-19 infections may go undetected and persist among stricken communities in countries with weak health systems, there needs to be: worldwide availability of medicines and vaccines treating COVID-19; provision for greater food security and ecosystem recovery; restoration of damaged rural agricultural and urban food production systems; and the revival of local ecosystem services. Failure to support such measures will be an immense injustice for future generations as well as for the most deprived communities of today.

## Thoughts on the wider implications of COVID-19

Human behaviour may change rapidly in times of crisis. People become more cautious about interaction with others. Some are highly conscious of social distancing, others say it does not matter. Some wear facial masks as a voluntary precaution, others refuse to do so, even where it is compulsory. There may be differences between both cities and countries. Financial Times journalist Gilliam Tett compared the strong sense of social pressure in New York with a more casual attitude in London, 66% of New Yorkers wearing masks in April compared to 25% in London (Tett 2020, p. 46). However, Londoners, perhaps more concerned about social distancing, were less likely to order takeaways than New Yorkers. Within the UK, a vivid contrast in behaviour persists between people who rush to beaches or attend illegal raves and leave tonnes of litter behind, and the thousands of volunteers who work hard to clean up their local beaches and country parks (Wollaston 2020, pp. 4-5). Such behavioural differences could be important in future transmission of the coronavirus and in managing the reduction in lockdown measures.

Responses to the pandemic differ across all scales from households and neighbourhoods to countries and continents. Reactions to extended or re-imposed lockdown measures in urban greenspaces range from acceptance to the provocative ignoring and deliberate staging of illegal crowd-pulling events. Young people congregate in urban greenspaces. People deliberately avoid wearing facemasks in places where they are required. Contrasts in guidance from central and regional governments lead to confusion. Confusion provides excuses for not doing what common sense really suggests should be done. Top-down responses and one-size-fits-all solutions, however good the modelling on which they are based, are unlikely succeed without insights that local knowledge and community understanding can bring. Internationally, states and multilateral organisations urgently need to devise a more effective approach to public health that integrates new national, regional, and international binding policies and initiatives (Milani 2020, p. 150). The current pandemic plays out much more quickly than the effects of human-induced climate change. However, the principle is the same: if you do not act before you can see the actual impact of the new coronavirus, it may be too late to stop its dissemination. Individuals, governments and international organisations must anticipate scenarios and act based on the precautionary principle so as to prevent the negative effects of the disease (Milani 2020, p. 150).

Public transport is avoided and car usage takes an upturn as soon as movement restrictions are lifted. Many people were seen walking for exercise in streets and urban greenspace in the first weeks of lockdown, but their numbers declined during June and July. By August 2020 in the UK, vehicular traffic and thus greenhouse gas emissions had returned to close to pre-pandemic levels. Perhaps this is an early indication that the impacts of COVID-19 on behaviour may be merely temporary and not the great shift that people expect. Yet, with the virus, everyone has been venturing into the unknown. The pandemic was still accelerating globally in its first phase but moving into second upsurges in many European countries in early August 2020. Further adaptation will be required. Continual adjustment is likely to be needed. The lessons from this health crisis are preparing, inducing and encouraging people to be ready for future global threats (Milani 2020), particularly the consequences of global heating.

For urban ecologists, the opportunity now exists to re-emphasise the value of nature in the city and to build on the apparent increase in community use of, and concern for, urban greenspaces. That opportunity has to be seised immediately and added to the calls from the UN and national governments to build a greener, healthier future. The values of urban ecosystem services and green infrastructure need to be high on the post-pandemic green recovery political agenda to meet the social desire to rebuild economies, increase employment and achieve a sustainable future.

## Footnotes

1 The comments received are all available on the UK Urban Ecology Forum website at (https://urbanecologyforum.org.uk/documents/Report_COVID19_returns_on_urban_ecologists.pdf), with another version at the Nature of Cities website (https://www.thenatureofcities.com/2020/07/04/how-is-covid-19-affecting-caring-for-and-researching-urban-ecology/).
